# Peak Eccentric Cycling Exercise and Cardiorespiratory Responses to Normobaric Hypoxia Versus Normobaric Normoxia in Healthy Adults: A Randomized, Controlled Crossover Trial

**DOI:** 10.3390/jcm14041151

**Published:** 2025-02-11

**Authors:** Carmen Wick, Esther Constam, Simon R. Schneider, Anna Titz, Michael Furian, Mona Lichtblau, Silvia Ulrich, Julian Müller

**Affiliations:** 1 Department of Pulmonology, University Hospital Zurich, 8091 Zurich, Switzerland; carmen.wick@usz.ch (C.W.); michael.furian@usz.ch (M.F.);; 2 Faculty of Medicine, University of Zurich, 8006 Zurich, Switzerland

**Keywords:** eccentric, eccentric cycle, eccentric bike, oxygen uptake, pulmonary rehabilitation, ergospirometry, hypoxia, normobaric hypoxia

## Abstract

**Background/Objectives**: Pulmonary rehabilitation clinics are traditionally located at higher altitudes (HAs), where lower PO_2_ reduces exercise capacity and blood oxygenation. Eccentric cycling exercise (ECC), with its lower cardiorespiratory demand compared to concentric cycling (CON), might therefore be a potential advantageous training modality at HAs, particularly for individuals with reduced exercise capacity. This study aimed to compare the cardiorespiratory responses of ECC while breathing normoxic versus hypoxic gas in healthy participants. **Methods**: This randomized, controlled crossover trial involved healthy participants performing CON in normoxia (FiO_2_ = 0.21), followed by two incremental ECC tests until 70–100% of peak exercise, one with normoxia and one with normobaric hypoxia (FiO_2_ = 0.15), in a randomized order. Oxygen uptake (V’O2) and additional outcomes were measured breath-by-breath. Endpoints were defined at rest, 50%, 70%, peak exercise, and isotime. The trial is registered on clinicaltrails.gov (NCT05185895). **Results**: Twelve healthy participants (age: 30 ± 11 years, six females) completed the study. During both interventions, V’O_2_ increased linearly with exercise intensity, with no significant differences between normoxic and hypoxic conditions. At peak exercise, SpO_2_ and peak work rate were significantly lowered by 5% (95%CI: 3 to 8%, *p* < 0.001) and by 22 W (95%CI: 8 to 36 W, *p* = 0.009) in hypoxia compared to normoxia. Other outcomes were unchanged. When comparing CON to ECC in normoxia, the mean differences in V’O2 increased with higher loads. **Conclusions**: This study demonstrated that V’O_2_ and other cardiopulmonary parameters remain unchanged when performing ECC in hypoxia compared to normoxia. Comparing CON to ECC in normoxia, participants achieved higher workloads and greater V’O_2_ consumption during CON compared to ECC at comparable watts, confirming the higher metabolic cost associated with CON. We identified that the optimal submaximal ECC intensities, with the highest difference in V’O_2_ between CON versus ECC, are around 40% of peak V’O_2_.

## 1. Introduction

Historically, rehabilitation centers are often located at moderate-to-high altitudes (HAs), as the mountain air is believed to offer significant health benefits. This practice continues today, supported by evidence that exercise at high altitudes induces several performance-enhancing changes in skeletal muscles, such as an increased capillary-to-fiber ratio [1,2,3,4], increased mitochondrial density [3,5], enhanced glycolytic capacity, and greater oxygen storage in muscles [6,7,8]. Further cardiovascular acclimatization such as the hypoxia-induced expression of the glycoprotein hormone erythropoietin collectively contributes to a rise in peak oxygen uptake (V’O_2_) [9,10,11] and improved exercise performance at low altitudes [12,13]. However, while these benefits of long-term acclimatization are notable, the reduced PO_2_ level at HAs produces physiological reactions, such as reduced exercise capacity, hypoxemia, and increased minute ventilation (V’E) [14,15,16,17]. Since cardiopulmonary rehabilitation centers are frequently located at HAs, these mechanisms are limiting, especially for patients with pre-existing cardiopulmonary diseases.

Eccentric cycling (ECC) has been shown to have significantly reduced cardiorespiratory and metabolic demands compared to traditional concentric cycling (CON) [18,19,20,21,22]. Specifically, for the same mechanical intensity, V’O_2_ during ECC is 60–80% lower than during CON at the same intensity [18,21,23], even at high exercise intensities [20,23]. A recent systematic review highlighted that ECC exercise is superior to CON exercise in improving muscle mass, knee extensor strength, and functional outcomes like six-minute walk distance in healthy volunteers and peak V’O_2_ in patients with cardiopulmonary diseases [24]. Exercise therapy is a crucial part of pulmonary rehabilitation, complementing pharmacological interventions [25,26]. Especially in the advanced stages of pulmonary disease, cardiopulmonary function is often so restricted that training intensity is too low for exercise on beneficial levels to sustain muscle mass [27]. ECC training presents a highly promising treatment option, especially for individuals with cardiopulmonary limitations who may struggle with traditional exercise methods at HA [18,28]. This approach has the potential to maximize the benefits of a pulmonary rehabilitation, especially at moderate-to-high altitudes.

Despite its potential, no studies have yet explored the feasibility and safety of ECC under hypoxic conditions, a critical factor for implementing this modality in HA rehabilitation settings. To address this gap, it is essential to first investigate ECC in healthy participants to establish preliminary evidence before extending research to clinical populations. To evaluate the feasibility and safety of ECC under hypoxic conditions, it is essential to first examine this in healthy participants, as no prior studies have investigated this effect. Therefore, this randomized clinical trial aimed to evaluate the cardiorespiratory responses to ECC under normoxia and normobaric hypoxia, providing preliminary data that could guide the development of tailored rehabilitation protocols for individuals with hypoxia-sensitive diseases.

## 2. Materials and Methods

### 2.1. Study Design

This was a randomized, controlled crossover study. Blinding was not possible due to the nature of the condition. The measurements took place at the University Hospital of Zurich, Switzerland, from August to September 2023.

### 2.2. Study Population

Healthy females and males between 18 and 80 years of age without any diagnosed cardiovascular diseases were included in this study. Exclusion criteria were the inability to follow the study protocol due to language problems, psychological disorders as well as neurological or orthopedic limitations with the inability to pedal on the ergometer, and participation in another clinical study that required active treatment. Two members of the study team recruited potential participants through word-of-mouth advertising. After participants signed the informed consent, they were enrolled and subsequently randomized by the same two team members. The study was approved by the regulatory authorities (KEK Zürich, BASEC-Nr. 2021-01312) and registered at clinicaltrials.gov (NCT05185895).

### 2.3. Study Procedure, Assessments, and Outcomes

All participants performed two study visits. During the first visit, all subjects underwent a spirometric test to assess lung volumes, which was followed by a CON incremental ramp exercise test until exhaustion to assess peak V’O_2_. After a 15-minute break, all participants completed a familiarization session on the eccentric ergometer for five minutes to prevent muscle soreness and damage [29]. The second visit occurred at least two days after the first visit. At visit 2, ECC under normoxia (FiO_2_ = 0.21) and hypoxia (FiO_2_ = 0.15, equivalent to an altitude of 2500 m) was performed in a randomized order with a wash-out time of two hours between the measurements.

For both interventions, the participants were seated on the eccentric ergometer and followed the same ramp protocol as in visit 1. After a 2-minute resting period, the participants cycled at 55–65 RPM with an increasing load, aiming to reach at least 70% of their peak CON load determined during the first visit. Participants could continue beyond 70% until exhaustion, but not exceed 100%.

CON was performed on a conventional ergometer (Ergoline-Ergoselect 200” Ergoline GmbH, Bitz, Germany), while ECC was performed on an eccentric ergometer (Cyclus 2 Recumbent EccentricTrainer, RBM elektronik-automation GmbH, Leipzig, Germany). During ECC, normobaric hypoxia was provided using the Everest Summit II altitude generator (Hypoxico Altitude Training Systems, Bickenbach, Germany). V’O_2_ and other ventilator outcomes were measured breath-by-breath with a metabolic unit (Geratherm Medical, Gschwenda, Germany) that was calibrated before each test [30]. The exercise tests were performed in accordance with the guidelines of the American Thoracic Society [31]. Every participant had the same protocol for both tests; however, protocols varied among participants according to their individual fitness levels, which were assessed according to the weekly hours of sport (<3 h: 6 W/20 s; 3–6 h: 8 W/20 s; 6–9 h: 10 W/20 s; 9–12 h: 12 W/20 s; >12 h: 14 W/20 s).

The primary outcome of the study was the mean difference in V’O_2_ at peak exercise between ECC under normoxia compared to that under hypoxia.

Secondary outcomes included the mean difference in V’O_2_ at rest, 50% and 70% of the individual maximal capacity, and other endpoints such as heart rate (HR), oxygen saturation (SpO_2_), carbon dioxide output (V’CO_2_), minute ventilation (VE), ventilatory equivalent for CO_2_ (VE/V’CO_2_), and oxygen pulse (O_2_Pulse). These variables were assessed at rest, 50%, 70%, and peak exercise. Additionally, dyspnea and leg fatigue were evaluated at peak exercise using the Borg CR10 scale.

### 2.4. Statistical Analysis and Sample Size

The sample size calculation was performed with a two-sided significance level of 0.05, a power of 0.8, a minimal clinical important difference of 0.6 L/min, and within a standard deviation of 0.4 L/min. According to these calculations, ten participants were needed. With an assumed dropout rate of 20%, a study population of twelve participants was aspired. Randomization was conducted using block randomization with randomly permuted blocks of size 2 using the package blockrand in R (version 4.3.0). The randomization sequence was developed by a member of the study team who was not involved in recruitment and measurement.

All data were summarized as means and standard deviations (SDs). To compare differences between normoxia and normobaric hypoxia, breath-by-breath data at each endpoint were averaged over 30 s. Endpoints were defined at rest, at 50%, at 70%, at peak exercise, and at isotime, while isotime compares tests with normoxia and hypoxia at identical times corresponding to peak exercise with the shorter tests. A linear mixed model was fitted with V’O_2_ as the dependent variable and condition as the fixed effect, while a random intercept accounted for individual differences in baseline V’O_2_. The Tukey method was used to adjust for multiple comparisons. Assumptions for linear mixed models, such as homogeneity and normality of residuals, were visually assessed using Tukey–Anscombe plots and Q–Q plots.

Additional outcomes were analyzed in a similar manner. All statistical analyses were conducted using R-studio Version 4.3.2, and a *p* of < 0.05 was assumed to reflect statistical significance.

## 3. Results

### 3.1. Baseline Characteristics

**Figure 1:**
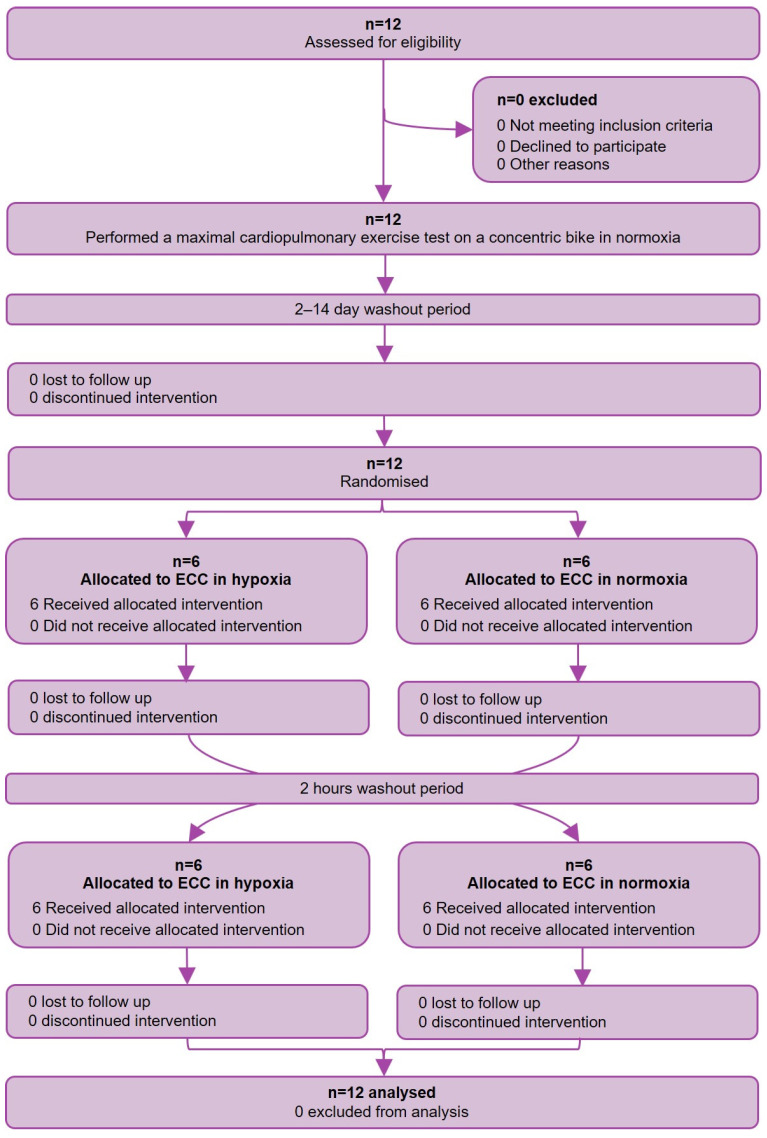
The flow chart of the included and analyzed patients. There were no dropouts.

Twelve participants were included into the study (six females). The mean ± SD age was 30 ± 12 years, and the body mass index was 22.3 ± 3.8 kg/m^2^. Baseline characteristics are listed in Table 1, and the study flow chart is shown in Figure 1.

Neither period nor carry-over effects were identified. In ECC under normoxia, participants reached 87 ± 8% of their normoxic CON workload, and one participant reached 100%. In ECC under hypoxia, participants reached 78 ± 16% of their hypoxic CON workload, and two participants reached 100%. Primary and secondary outcomes are presented in Table 2.

### 3.2. ECC Normoxia Versus Hypoxia

**Figure 2:**
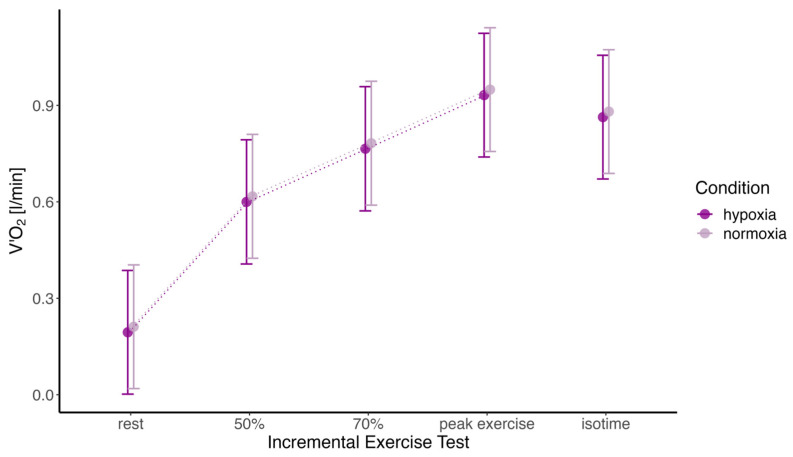
Mean oxygen uptake (V’O2) and standard deviation are presented for the different endpoints measured during normoxia and hypoxia. The terms “50%” and “70%” refer to 50% and 70% of each participant’s individual maximal capacity. “Isotime” corresponds to the maximum load each participant was able to sustain under normoxia and hypoxia.

During both interventions, V’O_2_ increased linearly with exercise intensity, with no statistically significant differences between normoxia and hypoxia at any endpoint (see Figure 2).

At rest, SpO_2_ showed a statistically significant difference between normoxia and hypoxia by 3% (95%CI: 2 to 5%, *p* = 0.001). The difference increased, with the largest disparity of 6% occurring at endpoints 70% (95%CI: 3 to 9%, *p* < 0.001) and peak exercise (95%CI: 3 to 9%, *p* < 0.001).

HR increased with exercise intensity during both interventions, remaining slightly higher in hypoxic ECC at all endpoints, though without reaching statistical significance. O_2_ pulse was lower in hypoxia at all endpoints; however, only the endpoint isotime showed a significant difference of 0.74 mL/min (95%CI: 0.01 to 1.47 L/min, *p* = 0.048).

Neither V’E nor V’E/V’CO_2_ varied significantly between the two interventions.

BorgCR10 scale for dyspnea and leg fatigue that were assessed at peak exercise were both slightly higher in hypoxia but neither showed statistically significant differences (95%CI: −1 to 1, *p* = 0.592; 95%CI: −2 to 0, *p* = 0.251).

### 3.3. Normoxic CON Versus Normoxic ECC

**Figure 3:**
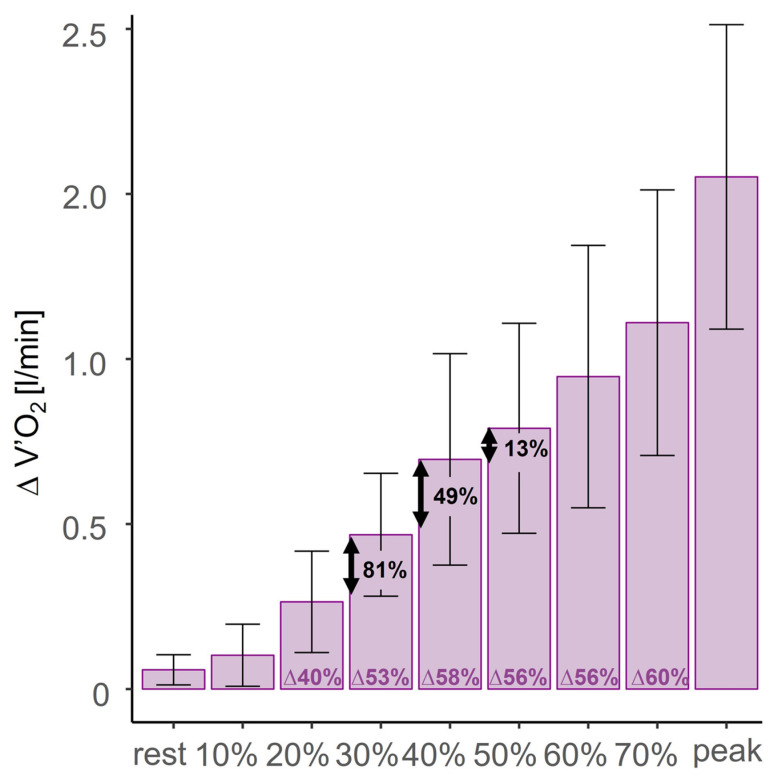
The absolute mean difference in oxygen uptake (V’O2) between concentric and eccentric cycling is reported alongside the standard deviation of these differences. The term “delta” represents the relative difference between concentric and eccentric cycling. The values 81%, 49%, and 13% indicate the relative increase in differences between successive steps, such as an 81% increase in the difference from 20% to 30%.

The mean differences in V’O_2_ increased with a higher load (see Table 2, Figure 3). The highest difference in V’O_2_ was observed at peak exercise, with a mean difference of 1.55 ± 0.46 L/min (95%CI: 1.28 to 1.82 L/min, *p* < 0.001). This corresponded to a 63% lower V’O_2_ in ECC than in CON. Apart from the increase in V’O_2_ difference between 70% and peak exercise, the greatest rise was observed from 30% to 40%, with a mean increase in V’O_2_ difference of 0.23 L/min. The mathematical calculation using fractions revealed that the highest dividend (absolute mean V’O_2_/relative mean power) of 1.75 occurred at 40%.

## 4. Discussion

The results of this study demonstrate that V’O_2_ and other cardiopulmonary parameters remain unchanged when performing ECC in a hypoxic condition equivalent to approximately 2500 m of HA compared to normoxia. During normoxic breathing, participants achieved higher workloads and greater V’O_2_ responses during CON compared to ECC, indicating higher cardiopulmonary and metabolic costs associated with CON. These findings reinforce the differences in metabolic demand between ECC and CON exercises, with ECC exercise requiring significantly less V’O_2_ despite comparable workloads [18,19,22].

The existing studies indicate that reduced PO_2_, due to normobaric hypoxia, physiologically leads to a decreased peak V’O_2_ in healthy individuals as well as in patients with cardiopulmonary diseases [17,32,33,34]. The observed reduction in V’O_2_ during ECC compared to CON aligns with the existing literature [18,19,22], reflecting the inherently lower cardiorespiratory demand of ECC. This submaximal nature of ECC explains why participants achieved similar percentages of their peak V’O_2_ in both normoxic and hypoxic ECC conditions, suggesting that ECC relies more on mechanical efficiency and less on oxygen-dependent metabolic pathways. Supporting these findings, prior research has shown that V’O_2_ remains stable during submaximal CON exercise (e.g., running at 55% of peak V’O_2_) in acute moderate hypoxia at altitudes up to 2800 m above sea level [17]. These results further highlight that, even when performed at the same intensity in terms of power output (watts), ECC training is less affected by hypoxia due to its lower cardiopulmonary demand. Additionally, dyspnea and leg fatigue scores were comparable between normoxic and hypoxic ECC, with dyspnea consistently rated lower than leg fatigue in both conditions. This suggests that ECC effectively reduces ventilatory demands, even under hypoxic conditions. These findings, consistent with previous studies [24,28,35], underscore ECC’s potential as a rehabilitative exercise modality, particularly for individuals prone to ventilatory limitations, and especially when rehabilitation is performed at HAs.

The observed reduction in SpO_2_ during hypoxia, which intensified with increasing exercise intensity, is consistent with the physiological response to lower PO_2_ levels. Desaturation is typically accompanied by increased ventilation and cardiac output to compensate for reduced oxygen availability; however, no significant changes in ventilation or HR were detected in this study. This could be attributed to the short exposure time to hypoxia (10–15 min), as full physiological acclimatization requires longer hypoxic exposure [36,37]. The peripheral chemoreceptors are oxygen-sensitive below a certain threshold of hypoxia. HR and cardiac output are the first to adapt to hypoxia, while ventilation acclimatization occurs over a longer time [37]. This may explain the small difference in HR and the similarity in ventilation observed in this study. Additionally, the type of hypoxia plays a role, with studies showing that acute hypobaric vs. normobaric hypoxia results in significantly lower SpO_2_, while in long-lasting hypobaric hypoxia, the difference between the two conditions diminishes [36,38]. Ventilatory parameters are also lower in hypobaric hypoxia, while cardiovascular variables appear similar between the two types [36,38]. These factors, along with the small sample size, may explain the absence of statistically significant differences in cardiorespiratory parameters between normoxic and hypoxic ECC.

In ECC, participants generated significantly less peak power compared to CON. Reason for this might be that ECC was novel for participants, and the lack of prior experience with this type of exercise likely influenced their ability to produce power effectively. ECC involves a unique motor pattern that differs from traditional concentric exercises, requiring greater neural coordination and unfamiliar movement dynamics. This learning curve may have compounded the challenges posed by hypoxia, further reducing power production. Additionally, acute submaximal ECC exercise in untrained individuals often induces micro-damage [19]. The lower peak power output in hypoxia compared to normoxia in ECC could be attributed to two different factors. The muscle micro-damage in ECC can lead to localized vascular dysfunction, making muscles more susceptible to deoxygenation [39], an effect likely exacerbated by hypoxia. Additionally, hypoxia is associated with reduced cognitive and executive function [40,41], as highlighted in a recent systematic review [41]. Since ECC exercise relies heavily on neural components [42], these pathways may be particularly vulnerable to the effects of hypoxia, further contributing to reduced power output. These findings underscore the interplay between muscular and neural mechanisms in hypoxia, though further research is needed to fully understand these mechanisms.

### 4.1. Minimal Intensity for Maximal Metabolic Cost Reduction in Normoxic ECC

The largest difference between normoxic CON and normoxic ECC in V’O_2_ was found at peak exercise. However, training at maximal effort requires high load capacity to withstand the strain and is therefore not efficient. Descriptive and mathematical analyses revealed that the minimal difference for maximal metabolic cost reduction is expected to be at 40% of the maximal power as visually demonstrated in Figure 3. Hence, training in that range seems to be the most efficient for cardiopulmonary cost. This knowledge can help future studies to test various training protocols since, to date, there is a lack of knowledge about which training parameters are the most beneficial. Even though this range seems to be the most efficient in terms of VO_2_ difference and performance, it is possible that other ranges may achieve better training effects. This must be further evaluated.

### 4.2. Implication for ECC in HA Rehabilitation Settings

Contrary to our initial hypothesis, hypoxia did not lead to a difference in V’O_2_ during ECC. This suggests that ECC effectively reduces ventilatory demands, even under hypoxic conditions. However, the drop in SpO_2_ observed during hypoxia could pose challenges for cardiopulmonary patients, particularly those with pre-existing conditions, as reduced oxygen availability may significantly impact their exercise tolerance. Since rehabilitation stays typically extend beyond two weeks, physiological acclimatization to HAs partially restores the SpO_2_ levels over time [43]. This needs to be further investigated in clinical cohorts to better understand its implications for long-term safety and efficacy.

Despite this, the study findings indicate that hypoxia did not significantly impact other cardiopulmonary parameters, suggesting that ECC remains a viable exercise modality even in HA settings. Additionally, the low dyspnea scores reported during ECC, even under hypoxic conditions, highlight its potential to minimize ventilatory strain. This unique combination of maintaining low cardiopulmonary demand and perceived exertion makes ECC a promising option for safe and effective rehabilitation programs for patients with cardiopulmonary diseases with reduced exercise tolerance, such as COPD, heart failure, or pulmonary hypertension, at HAs.

These findings, derived from a healthy and relatively young population, provide preliminary data for future studies. Future research should focus on investigating the effects of ECC in individuals with cardiopulmonary pathologies and extending the duration of hypoxic exposure to further evaluate its safety and efficacy. Optimal training protocols should also be explored, aiming to achieve maximal metabolic efficiency with minimal exertion, particularly around the identified 40% of individual maximal capacity.

### 4.3. Limitations

Blinding was impractical due to the setup, potentially leading to subjective bias in outcomes such as felt exertion. Additionally, participants were inexperienced with cardiorespiratory exercise testing, and the brief 10-min familiarization with ECC might have been insufficient, possibly impacting their performance [44].

Moreover, the study’s findings are limited in generalizability to the investigated population. Individuals with cardiopulmonary diseases were excluded, and the relatively young study population restricts applicability to older individuals or those with cardiopulmonary conditions. Despite these limitations, this study is the first to evaluate ECC under hypoxic conditions and to propose optimal training parameters for the ECC ergometer, offering a foundation for future research in this field.

## 5. Conclusions

This study demonstrated for the first time that V’O_2_, V’E/V’CO2, HR, V’E, O_2_Puls, dyspnea, and leg fatigue remain unchanged at peak exercise during ECC in normoxia compared to normobaric hypoxia. These findings highlight its potential as a promising exercise modality for cardiopulmonary patients in HA rehabilitation settings. Based on a healthy and young population, this study provides preliminary data for future research to explore ECC’s effects in cardiopulmonary patients, evaluate its safety during prolonged hypoxia, and identify optimal training protocols for maximizing metabolic efficiency at around 40% of individual maximal capacity.

## Figures and Tables

**Table 1:** Baseline characteristics are presented as mean (SD) or absolute values.

Characteristics	Value
Total Participants	12
Female	6
Male	6
Age [years]	30 (12)
Height [cm]	178 (10.1)
Weight [kg]	71.2 (11.1)
BMI [kg/m^2^]	22.33 (3.78)
Peak [V’O_2_]	2.54 (0.87)
Peak work rate [W]	269 (91)

**Table 2:** Columns under “Eccentric Cycling” compare outcomes between normoxia and hypoxia. Columns under “Normoxia” compare outcomes between concentric cycling (CON) and eccentric cycling (ECC). True values for estimates and SDs, and model values for CIs and *p*-values. * indicates statistical significance at alpha <0.05.

	Eccentric Cycling				Normoxia		
	Normoxia	Hypoxia	Normoxia–Hypoxia		CON	CON–ECC	
	Mean (SD)	Mean (SD)	Mean Change [95%CI]	*p*-Value	Mean (SD)	Mean Change [95%CI]	*p*-Value
**Rest**							
V’O_2_ [L/min]	0.21 (0.04)	0.20 (0.01)	0.01 [−0.04, 0.06]	0.619	0.27 (0.08)	0.06 [0.03, 0.09]	0.001 *
V’O_2_/kg [L/min/kg]	2.98 (0.50)	2.83 (1.44)	0.15 [−0.58, 0.90]	0.661	3.79 (0.84)	0.81 [0.45, 1.17]	0.001 *
V’E/V’CO_2_	42.3 (8.10)	48.1 (10.7)	−5.83 [−12.99, 1.33]	0.105	41.42 (8.74)	−0.89 [−4.78, 3.00]	0.650
HR [bpm]	63 (11)	65 (13)	−2 [−8, 6]	0.675	76 (9)	13 [7, 18]	<0.001 *
V’E [L/min]	10.3 (2.23)	11.8 (3.93)	−1.42 [−3.75, −0.91]	0.220	12.25 (3.25)	1.92 [0.21, 3.63]	0.043 *
O_2_Puls [mL/beat]	3.40 (0.97)	3.00 (1.39)	0.40 [−0.36, 1.16]	0.283	3.52 (0.92)	0.13 [−0.04, 0.29]	0.155
SpO_2_ [%]	97 (1)	94 (3)	3 [2, 5]	0.001 *	97 (1)	0 [−1, 0]	0.615
**50% of individual maximal capacity**							
V’O_2_ [L/min]	0.60(0.24)	0.65 (0.27)	−0.05 [−0.12, −0.03]	0.199	1.38 (0.47)	0.79 [0.6, 0.98]	<0.001 *
V’O_2_/kg [L/min/kg]	8.28 (2.36)	9.05 (2.92)	−0.77 [−1.86, 0.58]	0.289	19.58 (5.25)	11.34 [8.8, 13.88]	<0.001 *
V’E/V’CO_2_	33.5 (6.29)	35.9 (8.88)	−2.4 [−6.53, 1.58]	0.217	30.63 (5.31)	−3.43 [−5.86, −1]	0.015 *
HR [bpm]	90 (8)	97 (18)	−7 [−18, 2]	0.128	122 (14)	33 [24, 42]	<0.001 *
V’E [L/min]	21.3 (5.69)	24.0 (8.34)	−2.7 [−7.19, 1.79]	0.224	41.67 (12.07)	20.08 [13.49, 26.68]	<0.001 *
O_2_Puls [mL/beat]	6.65 (2.50)	6.53 (2.18)	0.12 [−0.44, 0.98]	0.434	11.10 (3.01)	4.49 [3.63, 5.35]	<0.001 *
SpO_2_ [%]	97 (1)	92 (4)	5 [3, 7]	<0.001 *	96 (2)	−1 [−2, 0]	0.095
**70% of individual maximal capacity**							
V’O_2_ [L/min]	0.77 (0.320)	0.81 (0.352)	−0.04 [−0.11, 0.08]	0.723	1.86 (0.62)	1.11 [0.87, 1.35]	<0.001 *
V’O_2_/kg [L/min/kg]	10.7 (3.35)	11.2 (3.67)	−0.5 [−1.75, 1.48]	0.862	26.46 (7.08)	16.00 [12.63, 19.37]	<0.001 *
V’E/V’CO_2_	33.3 (7.18)	34.9 (9.54)	−1.6 [−6.01, 2.19]	0.342	30.91 (5.37)	−2.74 [−5.95, 0.47]	0.110
HR [bpm]	100 (7)	107 (20)	−7 [−16, 3]	0.147	143 (15)	43 [34, 52]	<0.001 *
V’E [L/min]	26.0 (6.89)	27.7 (10.5)	−1.7 [−5.08, 2.59]	0.505	62.58 (15.30)	36.92 [29.02, 44.81]	<0.001 *
O_2_Puls [mL/beat]	7.67 (3.06)	7.50 (2.73)	0.17 [−0.47, 1.36]	0.322	12.90 (3.30)	5.30 [4.25, 6.35]	<0.001 *
SpO_2_ [%]	97 (1)	91 (5)	6 [3, 9]	<0.001 *	96 (2)	−1 [−2, 0]	0.045 *
**Peak exercise**							
Power [W]	235 (84)	213 (86)	22 [8, 36]	0.009 *	269 (91)	34 [18, 50]	0.001 *
V’O_2_ [L/min]	0.99 (0.51)	0.90 (0.38)	0.09 [−0.04, 0.22]	0.172	2.54 (0.87)	1.55 [1.28, 1.82]	<0.001 *
V’O_2_/kg [L/min/kg]	13.6 (5.70)	12.4 (4.35)	1.2 [−0.76, 3.14]	0.217	35.98 (9.95)	22.35 [18.44, 26.26]	<0.001 *
V’E/V’CO_2_	33.3 (11.9)	32.9 (7.80)	0.4 [−6.46, 7.26]	0.904	35.32 (4.09)	2.04 [−4.27, 8.35]	0.523
HR [bpm]	111 (15)	114 (20)	−3 [−11, 5]	0.459	167 (20)	55 [43, 68]	<0.001 *
V’E [L/min]	30.5 (10.4)	29.4 (12.0)	1.1 [−4.63, 6.79]	0.696	116.6 (38.6)	86.08 [66.43, 105.74]	<0.001 *
O_2_Puls [mL/beat]	8.60 (3.57)	7.68 (2.56)	0.92 [−0.12, 1.96]	0.081	13.20 (4.27)	5.00 [2.99, 7.66]	0.001 *
SpO_2_ [%]	96 (1)	91 (5)	5 [3, 8]	0.001 *	87 (20)	−9 [−20, 2]	0.115
Dyspnea [CR10]	3 (2)	4 (2)	−1 [−1, 1]	0.592	7 (2)	4 [3, 5]	<0.001 *
Leg fatigue [CR10]	6 (2)	7 (2)	−1 [−2, 0]	0.251	8 (2)	2 [1, 3]	0.011 *
**Isotime (individual maximal workload (in watts) that a participant was able to perform under both conditions)**							
Power [W]	213 (86)	213 (86)	-	-	213 (86)	-	-
V’O_2_ [L/min]	0.88 (0.44)	0.86 (0.38)	0.02 [−0.06, 0.10]	0.650	2.01 (0.964)	1.13 [0.77, 1.48]	<0.001 *
V’O_2_/kg [L/min/kg]	12.2 (4.88)	12.0 (4.40)	0.2 [−1.04, 1.51]	0.708	28.24 (11.8)	16.04 [11.03, 21.04]	<0.001 *
V’E/V’CO_2_	33.1 (7.50)	33.2 (7.48)	−0.1 [−4.42, 4.25]	0.969	38.73 (17.5)	5.63 [−10.08, 7.63]	0.783
HR [bpm]	107 (13)	114 (20)	−7 [−14, 0]	0.050	144 (28)	37 [23, 53]	<0.001 *
V’E [L/min]	29.7 (7.36)	29.8 (11.9)	−0.1 [−7.75, 7.59]	0.983	32.2 (4.69)	2.5 [−4.22, 2.33]	0.570
O_2_Puls [mL/beat]	8.12 (3.32)	7.38 (2.45)	0.74 [0.01, 1.47]	0.048 *	12.3 (4.15)	4.18 [2.82, 5.90]	0.002 *
SpO_2_ [%]	97 (1)	91 (5)	6 [3, 9]	<0.001 *	94 (3)	−3 [−4, −1]	0.011 *

## Data Availability

The datasets generated and analyzed during the current study are available from the author on reasonable request.

## Abbreviations

The following abbreviations are used in this manuscript:

BMI	Body mass index
CI	Confidence interval
CON	Concentric cycling
CR10	Clinical rating scale 10
ECC	Eccentric cycling
HA	High altitude
HR	Heart rate
O_2_	Oxygen
O_2_Pulse	Oxygen pulse
RPM	Repetitions per minute
SD	Standard deviation
SpO_2_	Oxygen saturation
V’CO_2_	Carbon dioxide output
V’E	Minute ventilation
V’E/VO_2_	Ventilatory equivalent for O_2_
V’E/V’CO_2_	Ventilatory equivalent for CO_2_
V’O_2_	Oxygen uptake
LD	Linear dichroism
